# Experimental Studies and Modeling of the Degradation Process of Poly(Lactic-*co*-Glycolic Acid) Microspheres for Sustained Protein Release

**DOI:** 10.3390/polym12092042

**Published:** 2020-09-08

**Authors:** Paolo Antonio Netti, Marco Biondi, Mariaenrica Frigione

**Affiliations:** 1 Interdisciplinary Research Centre on Biomaterials, CRIB, Università di Napoli Federico II, P.leTecchio, 80, 80125 Napoli, Italy; paoloantonio.netti@unina.it; 2 Department of Chemical, Materials and Industrial Production Engineering, Università di Napoli Federico II, P.leTecchio, 80, 80125 Napoli, Italy; 3 Dipartimento di Farmacia, Università di Napoli Federico II, Via D. Montesano 49, 80131 Napoli, Italy; 4 Department of Innovation Engineering, University of Salento, Via Arnesano, 73100 Lecce, Italy; mariaenrica.frigione@unisalento.it

**Keywords:** degradation of PLGA, kinetics of degradation, PLGA microspheres, drug delivery, mathematical modeling

## Abstract

In this study, poly(lactic-*co*-glycolic acid) microspheres (PLGA MS)for controlled protein release by double emulsion-solvent evaporation were produced and characterized for their morphological and technological features. MS autocatalytic degradation was described by a mathematical model based on a Michaelis and Menten-like chemical balance. Here, for the first time MS degradation was correlated to the advancement of MS degradation front with respect to the degraded radius, derived from mass loss experiments. The model can satisfactorily describe the kinetics of advancement of the degradation front experimentally derived for all MS formulations, especially when produced at higher PLGA concentrations.

## 1. Introduction

Synthetic and natural polymers normally degrade during their service-life, due to the exposure to different environmental conditions. Degradation is typically considered a negative event, since the performance, and the usefulness, of the altered polymers can be substantially impaired [1,2]. In other fields of application, however, a (controlled) polymer degradation can be advantageously exploited to release, for instance, drugs and fertilizers [3,4]. In particular, the delivery of drugs from systems based on degradable biomaterials proved to offer important advantages, such as controlled drug release kinetics, which correlates with limited oscillation of drug concentration in blood, a reduction of number of drug administration, and an overall improvement of therapeutic efficacy and patient compliance [5].

In this context, a pivotal biomaterial is poly(lactic-*co*-glycolic acid) (PLGA), which is a thermoplastic, random, synthetic copolymer made up of lactic (LA) and glycolic (GA) acid units. PLGA has been extensively studied for applications in controlled drug delivery and tissue engineering owing to its favorable properties, such as biocompatibility, non-toxicity, non-immunogenicity, mechanical resistance, and full biodegradability in vivo [6,7]. In an aqueous environment, PLGA chain undergoes hydrolytic attack to the ester bond and the polymer totally degrades into LA and GA, which are endogenous compounds normally metabolized through the Krebs cycle [8,9]. PLGA can be processed in many different shapes and sizes; furthermore, it is able to encapsulate, and subsequently release, molecules of any size [10].

The degradation kinetics of PLGA can be tailored by its molecular weight, LA/GA molar ratio (a higher content of LA leads to PLGA with a lower hydrophilicity, that absorbs less water and, thus, degrades slower), and presence or absence of capping [11,12,13]. A controlled degradation allows to engineer the release kinetics of encapsulated drugs in PLGA micro/nanodevices [14,15]. Inthis regard, PLGA has received approval by Food and Drug Administration (FDA) and European Medicines Agency (EMA) for human use, and it is the material of choice in the production of a wide array of drug delivery systems [16].

More in detail, PLGA-based microspheres (MS) are among the most successful drug delivery systems [15,17,18,19,20] both in lab and medicine [17], since they allow an effective control of drug kinetics [21]. Drug discharge is mainly governed by the complex interplay between drug diffusion within the polymeric matrix and the heterogeneous degradation of the polymeric matrix of MS. Release rates can be tailored by judiciously selecting PLGA features and formulation conditions [22,23,24], along with MS size/initial porosity, the type of drug and its radial distribution within MS [10,25,26]. The knowledge of the transport and physic-chemical processes that control the rate of drug release is fundamental for the rational design of MS.

The degradation of PLGA MS is triggered by hydrolysis of ester bonds and is catalyzed by acids. The process is generally heterogeneous since PLGA undergoes autocatalysis [27]. Indeed, the random hydrolysis of ester bonds causes the formation of new carboxylic groups along with a pH drop within MS matrix, thereby further catalyzing the hydrolysis of ester linkages. During this stage, PLGA molecular weight steadily decreases and, when it reaches the water solubility threshold (i.e., when nonamers are formed [28,29]), the insoluble-soluble transition of the polymer occurs. The soluble oligomers diffuse outwards according to a process controlled by degradation and solubility [28], causing mass erosion of the MS matrix [30], as well as the progressive increase of the void volume inside MS [30,31]. This complex balance results into the erosion of PLGA MS, which is favored in the internal sections of the devices, while occurring at a lower extent at the border of microspheres [32].

The objective of this work is the development and validation of a mathematical model ableto describe the heterogeneous bulk degradation of PLGA MS. In the last couple of decades, several models have been proposed aiming to describe drug release mechanisms from PLGA-based MS [33]. Actually, drug delivery is governed by a complex mechanism that can lead to monophasic or multi-phase (i.e., biphasic or triphasic) release [14]. During drug discharging from MS, manifold phenomena do occur, such as water absorption and hydrolysis, bulk/surface degradation, matrix erosion. In turn, these phenomena are strongly influenced by the parameters and conditions of the process, by the composition and by the physical-chemical properties of the system. The kinetics of the whole mechanism can be described employingdifferent theoretical, mathematical or computational models which can be roughly classified as diffusion-, surface erosion- and swelling-based models. More frequently, drug delivery from PLGA MS is modeled using Fick’s second law-based approaches. However, the applicability of the analytical solutions of this equation is somehow limited since it is based on the empirical calculation of several variables, primarily diffusivity. In order to expand the applicability of this model, the diffusion coefficient can be related to the variation of molecular weight as consequence of the degradation; the non-uniform distribution of the drug inside thepolymer can be also considered in the model [10,34].

Recently, Busatto et al. [25] developed a model that, simulating the homogeneous degradation of PLGA MS, was able to predict the reductions in molecular weight during the degradation process and to estimate the mass loss of the system. This model was subsequently extended to include also dissolution and diffusion of the drug in the PLGA matrix, inside and on the surface of MS [35]. Besides, this latter model was able to estimate the modifications in morphology during the degradationof MS possessing different sizes and to assess the effects of molecular weight andparticle dimensions on their degradation process. In another study [36], the influence of formulation variables and particle characteristics on the degradation kinetics of PLGA MS was examined in detail. Results revealed adegradation rate which was linearly increasing with MS size, thereby indicatingthat the autocatalysis of the PLGA was favored by the increasing size. Another approach was focused on drug release mainly controlled by diffusion within a time-evolving matrix, and described a molecular weight/time-dependent drug diffusivity within the degrading particle [37]. Furthermore, diffusion-control release has been taken into account to fit release data with semi-empirical models, such as Ritger-Peppas, Weibull, and Peppas-Sahlin, and a theoretical approach relying on Fick’s second law, aiming to correlate release kinetic parameters with PLGA molecular weight and MS formulation [38].

Nevertheless, to the best of ourknowledge, none of the models recently appeared in literature proposed to simulate the advancement of MS degradation front withthe measurements of the radius of the degraded spheres, as in the present study. In this work, model equations have been defined based on the autocatalytic mechanism of PLGA degradation kinetics, which has been described by a Michaelis and Menten reaction, following a previous publication [39]. The model takes into account the reaction between soluble acids and the polymer to define the rate of generation of soluble oligomers and monomers. To validate the model, three MS formulations loaded with rhodamine-labeled bovine serum albumin (BSA-Rhod), possessing comparable size, were produced and characterized, and their degradation in a phosphate-buffered saline buffer wasinvestigated. The developed model was, then, employed to study the effect of the formulation variable on the degradation/erosion kinetics of protein-loaded PLGA MS.

## 2. Materials and Methods

### 2.1. Materials

Equimolar uncapped poly(d,l-lactide-*co*-glycolide) (PLGA) was supplied by Boehringer Ingelheim (Ingelheim am Rhein, Germany) with the tradename (Resomer RG 504 H, Mw = 41.9 kDa, inherent viscosity of 0.5 dL/g). Bovine serum albumin, labeled with rhodamine as a fluorescent dye (BSA-Rhod, Mw = 66 kDa), was purchased from Molecular Probes Europe BV (Leiden, The Netherlands). Analytical grade dichloromethane (DCM) was provided by Carlo Erba (Milano, Italy). Tissue-Tek embedding medium was supplied by Sakura Finetek (Torrance, CA, USA). All other chemicals poly(vinyl alcohol, PVA, Mowiol 40–88; tetrahydrofuran, THF; polystyrene standards for GPC calibration; sodium hydroxide) from Sigma Aldrich (St. Louis, MO, USA) were used.

### 2.2. Microspheres Preparation

PLGA microspheres loaded with BSA-Rhod at the theoretical loading of 0.25% (0.25 mg of BSA-Rhod*per*100 mg of microspheres) were prepared by the double emulsion-solvent evaporation technique [23,40,41,42]. First, an internal aqueous phase was prepared by dissolving BSA-Rhod in 0.30 mL of water and poured into an organic phase composed of 3.0 mL of a PLGA solution in methylene chloride (10, 15 and 20% weight-to-volume (*w*/*v)*; correspondingly the formulations were named PLGA10, PLGA15, and PLGA20). The primary emulsion was generated by a high-speed homogenizer (Diax 900 equipped with a tool 6G, Heidolph, Schwabach, Germany) at 15,000× *g* rpm for 2 min. The double emulsion was obtained by adding the primary emulsion to an external aqueous phase made up of 30 mL of 0.5% (*w*/*v*) aqueous PVA and homogenizing for 1 min at 8000× *g* rpm (tool 10F). The organic solvent was, then, evaporated under magnetic stirring for 3 h at room temperature (MR 3001K, Heidolph, Schwabach, Germany) for MS hardening. Finally, MS were washed three times with distilled water by centrifugation at 9000× *g* rpm in a Universal 16R centrifuge (Hettich Zentrifugen, Tuttlingen, Germany) and lyophilized employing a Modulyo (Edwards, Burgess Hill, UK) for 24 h (0.01 atm, −60 °C). The obtained freeze-dried microspheres were stored at −20 °C.

### 2.3. Microspheres Characterization

#### 2.3.1. Microspheres Size and Size Distribution

The average size and size distribution of the produced microspheres were determined by analyzing a dispersion of lyophilized particles in 0.5% *w*/*v* of aqueous PVA by laser light scattering (employing a Coulter LS 100Q, Madison, WI, USA). The average size of MS was expressed as the mean volume diameter ± the standard deviation of the values collected from at least three independent lots.

#### 2.3.2. Protein Encapsulation Efficiency

The loading efficiency of BSA-Rhod in MS was calculated by dissolving the microparticles in a 0.5 N NaOH solution (0.5% *w*/*v*) under stirring for 24 h. The obtained solution was, then, centrifuged at 5000× *g* rpm and 4 °C, and analyzed by a spectro-fluorometric assay (employing a luminescence spectrometer LS 55, PerkinElmer, Hopkinton, MA, USA) using 96-well flat-bottomed plates (BD Falcon™, Becton, Dickinson &Co., Brentwood, TN, USA; λ_ex_ = 553 nm; λ_em_ = 577 nm). Prior to the analyses, the linearity of the relationship between fluorescence and concentration was assessed in the 0.25–10.0 μg/mL concentration range (r^2^ > 0.98). Results were averaged over three batches.

#### 2.3.3. Degradation Studies

The degradation of MS was studied by suspending the devices in a phosphate buffer saline solution, PBS (containing 120 mM of NaCl, 2.7 mM of KCl, 10 mM of phosphate salts, with a final pH of 7.4) (0.1% *w*/*v*), and incubating the suspensions at 37 °C on an undulating rocker platform (speed = 15× *g* rpm) (Stovall Life Science Inc., Greensboro, NC, USA). At scheduled time intervals, MS were centrifuged, washed three times, and lyophilized.

#### 2.3.4. Morphological Studies

The external and internal morphology of freeze-dried MS was analyzed by Scanning Electron Microscopy (SEM) (Leica S440, Wetzlar, Germany), after gold-sputtering each sample in high vacuum. To study the changes in time of internal morphology, the MS were dispersed in Tissue-Tek^®^ embedding medium and fixed in Cryomold^®^ (Sakura Finetek, Torrance, CA, USA) and cryosectioned (Accu-Cut™) at −24 °C. The thickness of each section was fixed at 10 μm.

To avoid the mechanical stress due to cross-sectioning, Confocal Laser Scanning Microscopy (CLSM) analyses were also performed by an LSM 510 Zeiss confocal inverted microscope equipped with a Zeiss 20×/3 NA objective lens (Carl Zeiss, Wetzlar, Germany; λ_ex_ = 543 nm; λ_em_ = 572 nm). CLSM was set at time zero with optimized laser power, pinhole aperture, detector gain and amplifier offset values, which were fixed during the degradation time frame. Virtual equatorial cross-sections of MS were observed in time aiming to observe the polymeric matrix unperturbed within the degradation medium. Contrast images were acquired for an optimized vision.

#### 2.3.5. Molecular Weight Measurements

The weight and number-average molecular weights of degrading MS were determined by gel permeation chromatography (GPC) experiments performed on a high-performance liquid chromatography (HPLC; Shimadzu, Kyoto, Japan) apparatus. This latter consists of an LC-10ADvp solvent delivery module equipped with a Rheodyne (R) syringe loading sample injector (model 7725i), a RID-10A refractive index detector and a SCL-10Avp system controller. Two Phenogel (R) columns (300 × 7.8 mm^2^) (Phenomenex, Torrance, CA, USA) with 500 and 5000. A pores were used in series as a stationary phase. As a mobile phase, tetrahydrofuran (THF) was used (flow rate: 1 mL/min). The columns were calibrated with polystyrene standards in the 400–70,000 Da molecular weight range. The natural logarithm of molecular weight was dependent on retention time according to a quadratic law (R^2^ > 0.99). The results were expressed as nondimensional molecular weight as a function of time, which follows a first-order kinetics [43,44].

#### 2.3.6. Mass Loss Experiments

The loss of mass of MS during PLGA degradation was gravimetrically determined. In detail, dried microspheres were suspended in PBS and incubated under mild agitation as described in Section 2.3.3. At predetermined time points, the samples were centrifuged (10 min, 5000× *g* rpm), and DCM was added to the supernatant. Subsequently, each sample was vigorously stirred for at least two hours and left to sit until the organic phase (in which the non-eroded PLGA was dissolved) and the aqueous phase separate. The aqueous phase was carefully removed and replaced five times with the same amount of demineralized water to remove PBS salts, while the organic phase was allowed to evaporate by magnetic stirring for 24 h at room temperature. Then, the samples were freeze dried, and the vials were weighed again. The weight of the PLGA residue was calculated by subtraction. To assess the feasibility of the gravimetric tests, the method was carried out with known amounts of PLGA dissolved in DCM, emulsified with bidistilled water and lyophilized. In all cases, the known PLGA masses were unchanged within <4% error after freeze-drying. The vials were also weighted in the presence of fixed amounts of bidistilled water (1–15 μL) to assess the possibility to discriminate the polymer mass loss. A linear correlation was found out between the measured net weight and the known amounts of water (R^2^ > 0.99).

## 3. Mathematical Modeling

Protein release from PLGA MS is governed by an autocatalytic polymer degradation mechanism, and it can be schematized as follows: (i) initially, water intrudes in the device through its micro/macropores, and subsequent matrix plasticization occur [45]; (ii) hydrolytically initiated PLGA degradation at chain backbone takes place and acidic by-products are formed; (iii) the produced acids further catalyze polymer degradation and enhance the generation of unstable, insoluble intermediate esters which, in turn, undergo hydrolysis, thus favoring the formation of more soluble acidic degradation products. Reactions are coupled with diffusion-controlled mass transfer processes involving water, acid generator (i.e., the polymer), acids, and the protein to release. During degradation, the molecular weight steadily decreases, while the device mass loss starts when polymer molecules reach a threshold molecular weight below which PLGA is soluble [28,29].

The model has been developed considering spherical symmetry and an invariant geometry during PLGA degradation/protein unloading. Protein solubilization and release are governed by PLGA degradation which, in turn, is promoted in the MS center due to polymer autocatalytic degradation mechanism; the latter causes a decrease in pH and a more extensive polymer degradation in the central regions of the PLGA microspheres. Starting from these hypotheses, the bulk degradation of the MS has been modeled considering the existence of a degradation front moving outwards from MS center during the PLGA degradation/protein release. In the degraded MS inner region, the polymer undergoes the insoluble-soluble phase transition.

In spherical coordinates, radii were non-dimensionalized with respect to microsphere average MS radii. Thus, letting *ρ* be the non-dimensional radius, it results (Equation (1)):

(1) $$\rho =\frac{r}{R_{MS}}$$

where: *r* is the generic radius within MS, and *R_MS_* is the average radius for each MS formulation. Therefore, the position of the degraded front (i.e., *ρ*_DEG_) can be described as a function of time and has been modeled as next described. Neglecting the initial hydration phase, the following reaction scheme can be hypothesized (Equation (2)):

(2) $$A+P\overset{k_{1},\;\;\;k_{-1}}{↔}AP\overset{k_{2}}{\to }A+S$$

where: *A* is the soluble acid; *P* is the non-degraded polymer acting as a substrate; *AP* is the partially degraded, insoluble polymer; *S* is the generated soluble oligomer/monomer; *k*_1_, *k*_−1_, and *k*_2_ denote the rate constants of the forward, backward, and final degradation reactions, respectively.

Equation (2) formally resembles enzymatic degradation and can be described by a Michaelis and Menten-like equation [46,47]. Under the approximation of quasi-steady state, it is (Equation (3)):

(3) $$k_{1}\left[A\right]\left[P\right]=\left(k_{-1}+k_{2}\right)\left[AP\right]$$

Letting [*A*]_0_ be the initial acid concentration, it results [*A*] + [*P*] = [*A*]_0_. After substituting the latter in Equation (3), it follows (Equation (4)):

(4) $$\left[AP\right]=\frac{k_{1}{\left[A\right]}_{0}\left[P\right]}{k_{1}\left[P\right]+k_{-1}+k_{2}}$$

The rate of soluble product generation can be expressed as (Equation (5)):

(5) $$\frac{d\left[S\right]}{dt}=k_{2}\left[AP\right]=\frac{k_{1}k_{2}{\left[A\right]}_{0}\left[P\right]}{k_{1}\left[P\right]+k_{-1}+k_{2}}$$

Dividing Equation (5) by *k*_1_, and introducing a constant *K_M_* = (*k*_−1_ + *k*_2_)/*k*_1_ and an initial reaction rate *R*_0_ = *k*_2_[*A*]_0_, it follows (Equation (6)):

(6) $$\frac{d\left[S\right]}{dt}=\frac{R_{0}\left[P\right]}{K_{M}+\left[P\right]}$$

*K_M_* is a characteristic constant that estimates the polymer-acid affinity as it accounts for the ratio between polymer-acid disappearance versus generation. Indeed, low *K_M_* values indicate high affinity, i.e., that the generation of soluble products approaches the maximum rate in shorter times [48].

Letting *m_S_* and *MW_S_* be the mass and the molecular weight of the soluble products, respectively, and *V_MS_* the volume of a single MS, it results (Equation (7)):

(7) $$\left[S\right]=\frac{m_{S}}{V_{MS}MW_{S}}$$

It must be underlined that PLGA MS did not exhibit any swelling during protein release (data not shown); therefore, the radius has been considered constant. Furthermore, *MW_S_* is assumed to be basically constant during PLGA degradation. Thus, from Equation (7), it is derived (Equation (8)):

(8) $$\frac{d\left[S\right]}{dt}=\frac{1}{V_{MS}MW_{S}}\frac{dm_{S}}{dt}$$

Substituting the previous Equation (8) in (6) one, it is obtained (Equation (9)):

(9) $$\frac{dm_{S}}{dt}=V_{MS}MW_{S}\frac{R_{0}\left[P\right]}{K_{M}+\left[P\right]}$$

Concerning the polymer concentration, it is (Equation (10)):

(10) $$\left[P\right]=\frac{m_{P}}{V_{MS}MW_{P}}$$

where: *m_P_* and *MW_P_* are the time-dependent mass and the number-average molecular weight of PLGA, respectively. After substituting thelatter in (9), it is:

(11) $$\frac{dm_{S}}{dt}=V_{MS}MW_{S}\frac{R_{0}m_{P}}{K_{M}V_{MS}MW_{P}+m_{P}}$$

The following non-dimensional variables can be defined as follows (Equations (12)–(14)):

(12) $$\mu_{S}=\frac{m_{S}}{m_{MS,0}}$$

(13) $$\mu_{P}=\frac{m_{P}}{m_{MS,0}}$$

being: *μ_S_* + *μ_P_* = 1:

(14) $$\omega_{P}=\frac{MW_{P}}{MW_{P,0}}$$

In Equations (12)–(14): *m_MS_*_,0_ and *MW_P_*_,0_ are the mass of a single microsphere and the PLGA molecular weight at time zero, respectively; *μ_S_* and *μ_P_* are the non-dimensional mass of soluble products and of the residual polymer, while *ω_P_* is the non-dimensional polymer weight normalized with respect to its initial value.

Substituting Equations (14)–(16) into (11), and rearranging this latter equation, it follows (Equation (15)):

(15) $$\frac{d\mu_{S}}{dt}=\frac{B_{0}\left(1-\mu_{S}\right)}{\Psi \omega_{P}+1-\mu_{S}}$$

where *B*_0_ and ψ are adjustable parameters, defined as (Equations (16) and (17)):

(16) $$B_{0}=\frac{V_{MS}MW_{S}R_{0}}{m_{MS,0}}$$

(17) $$\Psi =\frac{K_{M}V_{MS}{MW}_{P,0}}{m_{MS,0}}$$

Furthermore, it is (Equation (18)):

(18) $$\omega_{P}=\exp\left(-k_{\deg}t\right)$$

where: *k*_deg_ is the degradation constant of the polymer; *ω_P_* was estimated from data published in a previous work [23], taking into account that the ratio between PLGA average weight and number average molecular weight at time *t* and time zero followed comparable decreasing kinetics (data not shown). It must be underlined that it was not necessary to separately define the time trends for the number average molecular weight and the weight average molecular weight in the present work. Indeed, GPC results published in [24] showed that the kinetic degradation constants were all of the same order of magnitude, regardless of the formulation. By virtue of this result, *ω_P_* expressed the overall molecular weight, normalized with respect the initial value. Correspondingly, an overall degradation constant, *k*_deg_, has been introduced here. Experimental observations showed that the polymer loss (i.e., generation of soluble acids) takes place after an induction time *t_ind_*, defined similarly to a previous work [49], which can be here described as the time necessary for PLGA to produce a significant amount of soluble oligomers. Accordingly, Equation (15) can be modified by using Heaviside step function [23,40], as expressed in Equation (19):

(19) $$\frac{d\mu_{S}}{dt}=\frac{B_{0}\left(1-\mu_{S}\right)}{\Psi \omega_{P}+1-\mu_{S}}H\left(t-t_{ind}\right)$$

To account for the continuous (non-discrete) transition of *μ_S_* in time, Heaviside function has been mollified as shown in the following Equation (20):

(20) $$\frac{d\mu_{S}}{dt}=\frac{1}{2}\frac{B_{0}\left(1-\mu_{S}\right)}{1+\Psi \omega_{P}-\mu_{S}}\left\{\tanh[\alpha (t-t_{ind})+1\right\}$$

being the initial condition (Equation (21)):

(21) $$\mu_{S}\left(0\right)=0$$

The constant *α* was introduced to define mollification radius, and its value was optimized to 2.5. The degraded non dimensional radius can be easily derived. Assuming that the density of PLGA is constant during degradation, Equation (14) can be re-written as (Equation (22)):

(22) $$\mu_{P}=\frac{V_{P}}{V_{MS}}=1-R_{f}^{3}\left(t\right)$$

Hence, (Equation (23)):

(23) $$R_{f}\left(t\right)=\sqrt[{3}]{\mu_{S}}$$

Overall, the kinetics of the degradation in time has been calculated through Equations (18), (20), and (23), using *B*_0_, *t_ind_*,and Ψ as adjustable parameters.

## 4. Results and Discussions

### 4.1. Microsphere Properties

Similarly to the results published in previous publications [23,40], particles with uniform diameters (slightly higher than 20 μm) and a protein encapsulation efficiency > 93%) were obtained for all the formulations, reported in Table 1.

**Figure 1:**
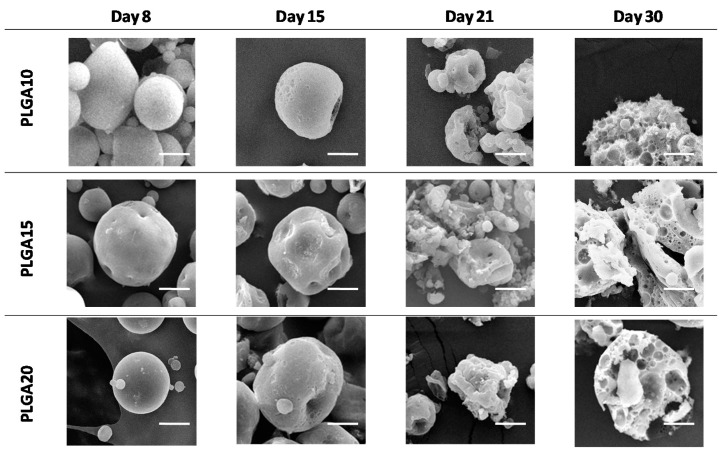
SEM micrographs of degrading MS. Progressive loss of structural integrity in time has been evidenced. The bar is 10 m.

**Figure 2:**
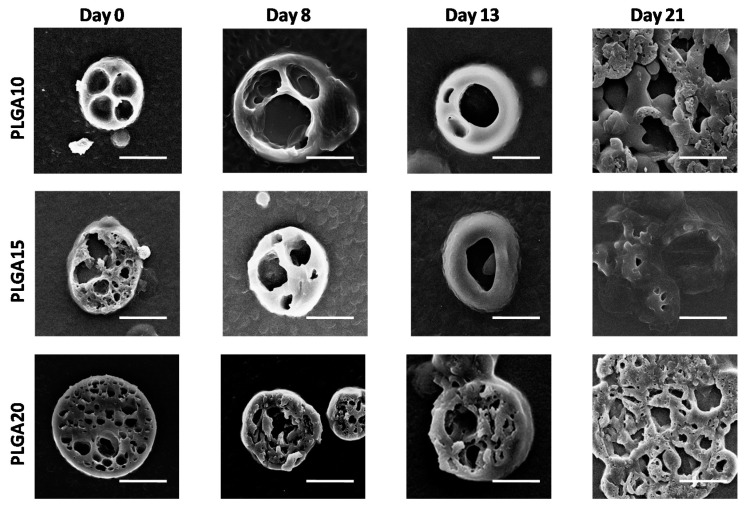
SEM images of cross sections of degrading MS. The formation of large pores within MS bulk in time is evidenced. The bar is 10 m.

The external aspect of particles was basically the same for all MS formulations (see Figure 1), while MS cross-sections showed that the formulation strongly affects the internal architecture of the devices (as noticed in Figure 2). Indeed, the internal aqueous pores were larger for PLGA10 MS and progressively smaller for PLGA15 and PLGA20 formulations. Actually, increasing PLGA concentrations are associated to a greater viscosity of the organic phase and, consequently, to a progressively hampered coalescence of the aqueous droplets [50].

SEM images, reported in Figure 1, clearly show that, regardless of the formulation variable, MS maintain their integrity up to approximately two weeks of degradation and then progressively collapse.

SEM observations conducted on sectioned MS (illustrated in Figure 2) provide further details and indicate that the external sections of the devices suffer limited effects during degradation up to 13 days. After 21 days, however, the sections of the MS appear collapsed on each other, probably due to the extensive bulk degradation of MS, which reduces their mechanical properties, thereby leading to MS damage during sectioning procedure.

To avoid the mechanical insult due to the blade during sectioning, also CLSM observations were performed on degrading MS. In particular, contrast images were taken and they showed that, over time, the polymer matrix becomes progressively looser within 30 days.

**Figure 3:**
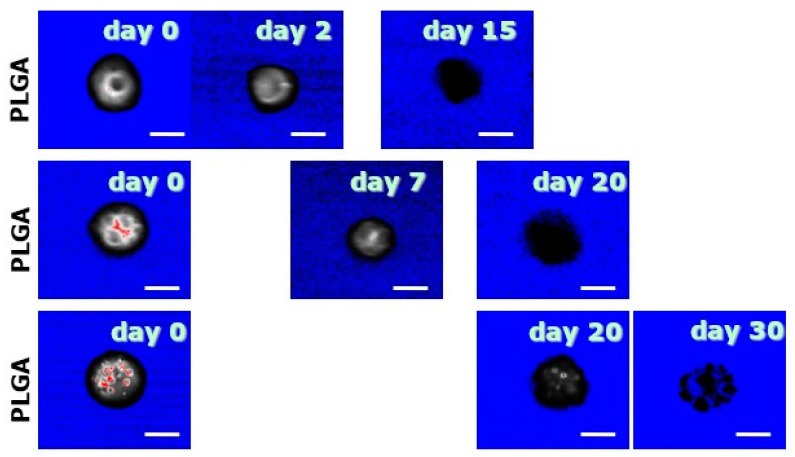
CLSM images of virtual cross-sections of MS. The progressive disappearance of the polymeric matrix is here shown. The bar is 10 m.

Figure 3 shows the CLSM images displaying the virtual sections of MS during degradation in the absence of the mechanical stress related to the blade during sectioning. The visual comparison with Figure 2 shows how, during their degradation, MS progressively lose fluorescence due to the release of BSA-Rhod [23,32,40]; at the same time, PLGA matrix progressively vanishes, still keeping the degrading MS an overall spherical geometry.

### 4.2. Determination of Microsphere Mass Loss and Degraded Radius

**Figure 4:**
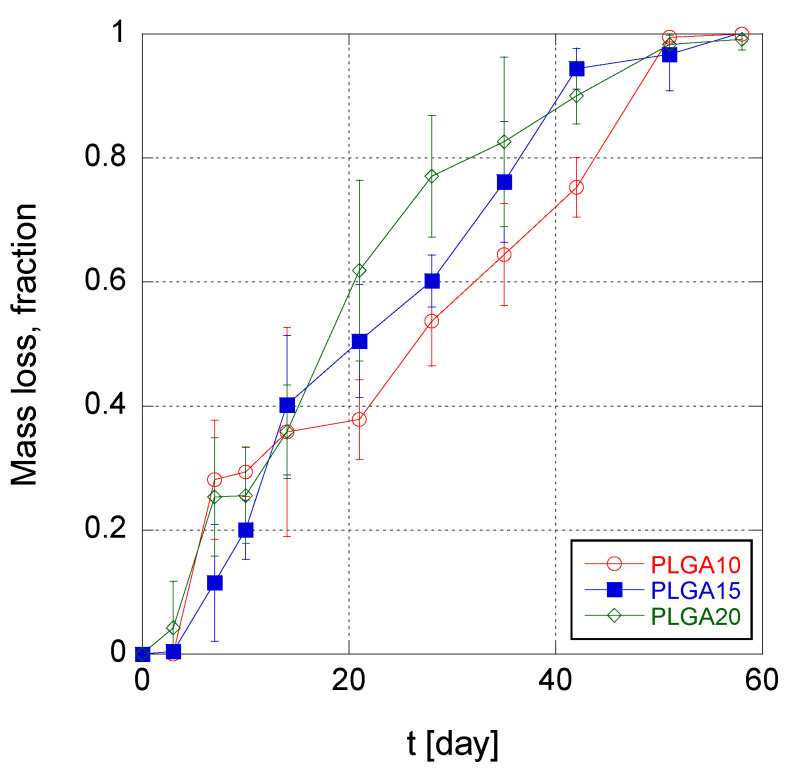
Time trend of mass loss of poly(lactic-*co*-glycolic acid) (PLGA) MS.

The trend of MS mass loss during time is illustrated in Figure 4 for the three different MS formulations. It can be noticed that, in the first 3–7 days, the mass loss was irrelevant, irrespective to the formulation. This indicates that water intrusion into MS takes place with a similar rate in the three produced MS formulations, and furthermore confirms that the erosion of PLGA MS is triggered when the molecular weight of the PLGA reaches the solubility threshold (about 1100 Da [51]). For longer times, i.e., from 2 to 6 weeks of degradation, the erosion rate of MS was found to be weakly increasing with increasing PLGA concentration in the organic phase of the emulsion. This suggests that an increasing polymer concentration within the MS matrix causes greater diffusion obstacles for the produced oligomers. Consequently, autocatalysis is favored in the long term for MS PLGA20 over PLGA15 and PLGA10 formulations.

### 4.3. Model for MS Degradation and Validation with Experimental Data

Generally speaking, kinetics models able to describe the mechanisms taking place during chemical-physical processes are considered of invaluable utility since they allow to predict the degree of advancement of the processes. In the present work, the degradation rate of PLGA MS was modeled as a function of the main chemical phenomena occurring during release process and of the formulation of the system under analysis. In this way, mathematical modeling in principle allows to properly modify the process conditions and the formulation composition in order to control the release mechanism, adapting it to specific requirements (e.g., optimizing process times/rates).

**Figure 5:**
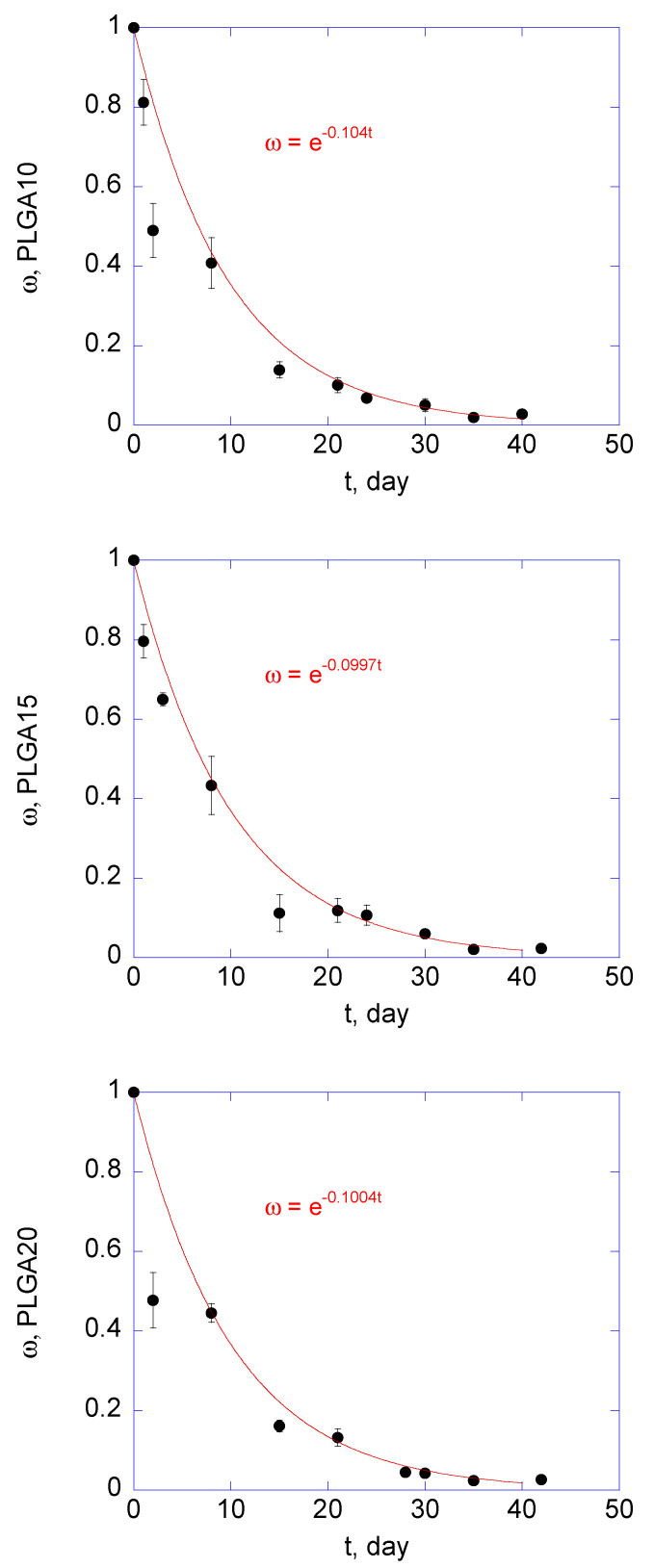
Time trend of non-dimensional molecular weight of PLGA10, PLGA15, and PLGA20 MS after suspension in phosphate buffer saline solution (PBS) (pH = 7.4). Experimental data were fit to Equation (19) (solid lines: fitting results).

In this work, a Runge-Kutta 4th order method was employed to run the model, using *B*_0_, *t_L_*, and Ψ as adjustable parameters. Model equations were validated using the radius of the propagation front for the three formulations, calculated by Equation (22). Equation (18), describing the time evolution of the overall, nondimensional molecular weight of PLGA, was independently adjusted to experimentally determined molecular masses of PLGA, using *k*_deg_ values equal to 0.104, 0.0997, and 0.1004 day^−1^ for PLGA10, PLGA15, and PLGA20, respectively. Experimental values and numerical simulations are shown in Figure 5.

**Figure 6:**
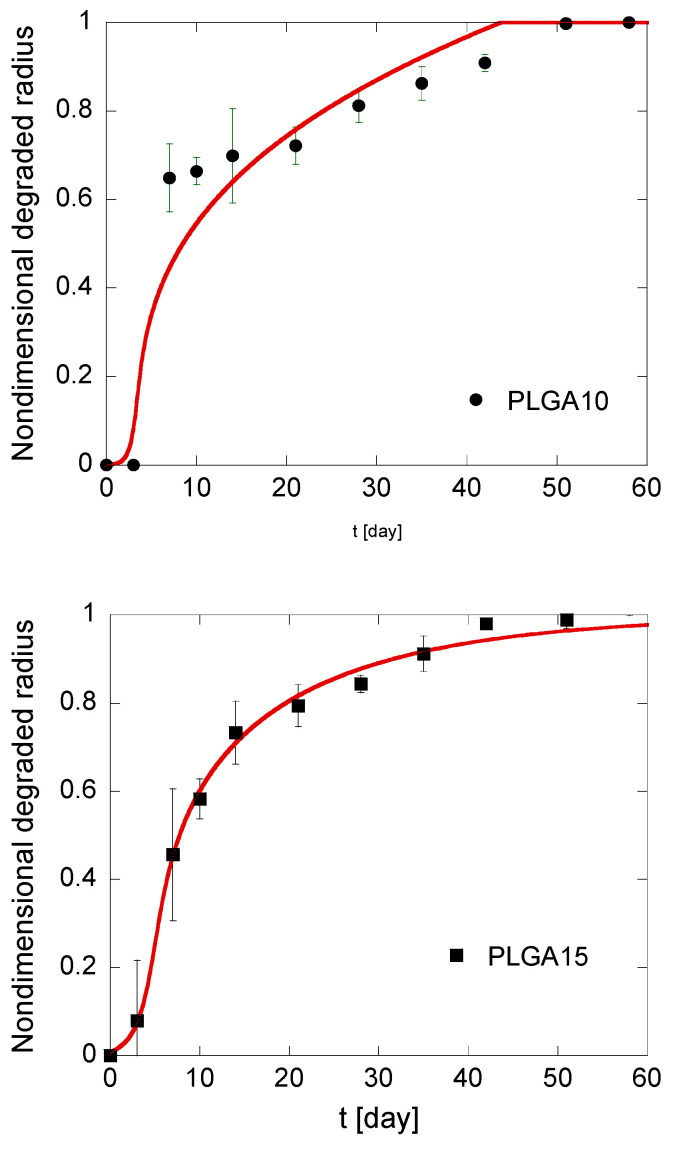

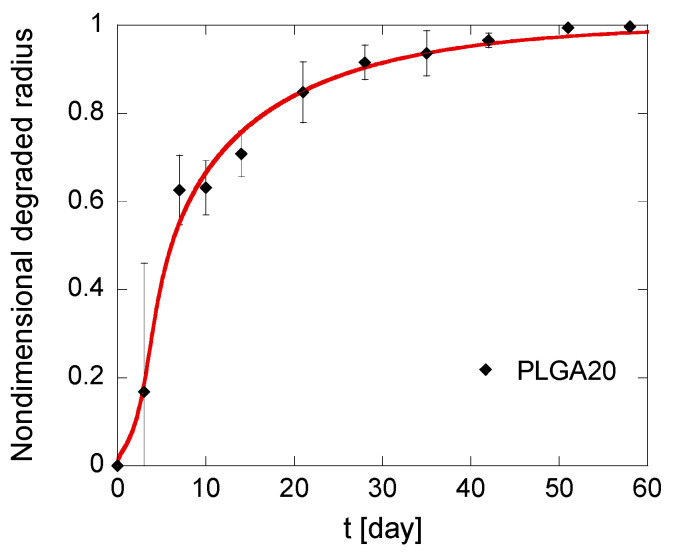
Kinetics of the degradation front of PLGA MS in different compositions. The model simulations, fitting the experimental data reported in black, are represented by solid red lines.

*B*_0_, as defined in Equation (16), accounts for the initial degradation rate, i.e., initial acid concentration, while Ψ, which is established in Equation (17), can be regarded as a measure of the stability of the acid-polymer complex. The results of the best fitting are reported in Table 2 and illustrated in Figure 6. From the observation of the data reported in Table 2, it results that *B*_0_ is slightly increasing with the PLGA concentration in MS formulations. Actually, due to autocatalysis, acid-polymer substrate is less stable because a tighter MS structure efficiently traps diffusing species, including soluble oligomers, thus promoting PLGA autocatalytic degradation. It must be underlined, however, that these differences are slight and can be grasped with some difficulty. The values of Ψ do not show a trend with polymer concentration in the organic phase of the emulsion and are far lower than unity. This is consistent with the negligible acid amount in MS before degradation starts. In particular, low Ψ values suggest the formation of a relatively stable acid-polymer complex, which is slowly transformed into soluble products.

Induction times are around 3 days for all formulations, which is consistent with the observation that MS mass loss occurs only when the first soluble oligomers are formed, i.e., when polymer molecular weight drops below the hydrosolubilization threshold [51]. In particular, the calculated *t_ind_* is lower for PLGA20 MS, thereby confirming that autocatalysis is slightly promoted at a high PLGA concentration. It must also be underlined that the fitting is better for PLGA15 and PLGA20 formulations compared to PLGA10 formulation. This can be reasonably ascribed to a high risk of error for this latter formulation. Indeed, the gravimetric method used to calculate the residual MS mass and, consequently, the degraded radius, is based on tiny differences in residual mass of MS. Autocatalysis is slightly promoted for PLGA15 and PLGA20 formulations, and this correlates to higher mass differences for short degradation times, when the mass loss are negligible compared to the initial MS mass. This suggests that the model is more accurate for PLGA-based MS produced by emulsion methods at high polymer concentration in the organic phase.

## 5. Conclusions

In this work, a mathematical model describing the heterogeneous degradation of PLGA MS has been developed. The effect of the concentration of the polymer in the organic phase used for the production of MS was investigated. The model, starting from the normalized experimental data of PLGA molecular weight, was run to provide an estimate of the radius of the degraded sphere (*R_f_*), which describes the advancement kinetics of MS degradation front.

The model was able to fit the experimental values of *R_f_* and was used to study the effect of the formulation variable on the degradation kinetics of the MS produced. The model can also be used to compare the advancement kinetics of the degradation front with the protein release kinetics already studied in [22,32,40].

Future developments of the model may deal with the comparison with the local protein concentration profiles in the MS PLGA during the degradation/release of the devices.

## Figures and Tables

**Table 1:** Composition and main characteristics of the produced microspheres (MS).

	PLGA in the Organic Phase (% *w*/*v*)	Mean Size (μm)	Encapsulation Efficiency (%)
PLGA10	10	24.3 ± 1.3	96.7 ± 4.7
PLGA15	15	23.0 ± 4.1	97.0 ± 2.0
PLGA20	20	24.1 ± 3.0	93.9 ± 6.5

**Table 2:** Best-fit parameters of the model (reported in Equation (19)) used to simulate propagation kinetics of the degradation front. Standard deviations (SD) of the parameters were calculated over three repeats.

	(*B*_0_ ± SD)∙10^2^ (day^−1^)	(Ψ ± SD)∙10^5^	*t_ind_* ± SD (day)
PLGA10	2.48 ± 0.36	2.69 ± 0.62	3.42 ± 0.43
PLGA15	2.72 ± 0.37	4.32 ± 1.23	3.40 ± 0.68
PLGA20	3.46 ± 1.28	1.59 ± 2.98	2.91 ± 0.09
